# Perceptions of the Three Dietary Patterns of the 2020–2025 United States Dietary Guidelines Among African American Adults After a 12-Week Randomized Intervention Trial to Reduce Type 2 Diabetes Risk: A Qualitative Study

**DOI:** 10.3390/nu17213453

**Published:** 2025-10-31

**Authors:** Halide Zeynep Aydin, Nkechi Okpara, Kelli E. Dubois, Mary M. Jones, Jessica Carswell, Sara Wilcox, Daniela B. Friedman, Angela D. Liese, Gabrielle Turner-McGrievy

**Affiliations:** 1 Department of Nutrition and Food Studies, College of Public Health, George Mason University, Fairfax, VA 22030, USA; 2 Department of Family and Consumer Sciences, California State University, Long Beach, CA 90840, USA; nkechi.okpara@csulb.edu; 3 Prevention Research Center, Arnold School of Public Health, University of South Carolina, Columbia, SC 29208, USA; kdubois@email.sc.edu (K.E.D.); wilcoxs@mailbox.sc.edu (S.W.); 4 Department of Health Promotion, Education, and Behavior, Arnold School of Public Health, University of South Carolina, Columbia, SC 29208, USA; jonesmary@sc.edu (M.M.J.); dfriedma@mailbox.sc.edu (D.B.F.); brie@sc.edu (G.T.-M.); 5 Department of Exercise Science, Arnold School of Public Health, University of South Carolina, Columbia, SC 29208, USA; 6 Department of Epidemiology and Biostatistics, Arnold School of Public Health, University of South Carolina, Columbia, SC 29208, USA; liese@sc.edu

**Keywords:** dietary guidelines, African Americans, nutrition, dietary patterns, qualitative research

## Abstract

**Background:** The United States Dietary Guidelines (USDG) form the basis of federal nutrition programs/policies for Americans. There has been little work to ensure that the presentation of the USDG are culturally acceptable and relevant for African Americans (AAs). This study aimed to explore the acceptability and perceptions of a randomized intervention among AA adults adhering to unmodified dietary patterns outlined in the USDG (Healthy US, Mediterranean, Vegetarian). **Methods:** Qualitative focus groups were conducted with participants from a USDG-based intervention, the Dietary Guidelines: 3 Diets study (DG3D). Six focus group discussions were conducted with AA adults in the Southeastern US in December 2021, after completion of the 12-week DG3D intervention. Verbatim transcripts were coded thematically and analyzed in Nvivo12 using an iterative constant comparative method. **Results:** Participants (*n* = 42; median age 52 years, range 26–65, 16 Healthy US, 17 Mediterranean, 9 Vegetarian) shared their perspectives and experiences adopting USDG dietary patterns and reported barriers and facilitators to adopting dietary change. Discussions elicited insights into the cultural relevance of the USDG and dietary intervention. Participants also described their recommended changes to the USDG-based intervention to enhance program implementation and successful dietary change. **Conclusions:** Study findings suggest that adaptations to the USDG dietary patterns are needed to ensure cultural relevance for AA adults in the US. This study can inform the development of culturally relevant dietary guidelines and intervention programs.

## 1. Introduction

The United States Dietary Guidelines (USDG) are developed in a collaborative effort between the United States Department of Agriculture (USDA) and the United States Department of Health and Human Services (USDHHS). The USDG provide recommendations to form policy, health education, and health promotion as well as industry and community-based intervention efforts to optimize the health of Americans [1]. The USDG are informed by the latest research in nutrition science and updated every five years, with the current guidelines reflecting the 2020–2025 period.

The 2020–2025 USDG emphasize foods from the five food groups (fruits, vegetables, grains, proteins, and dairy) as a foundation for a healthy diet. Included are recommendations for limiting saturated fats, added sugars, sodium, and alcohol. The guidelines emphasize combinations of food and beverages, rather than individual food items, that form eating patterns. Three specific dietary patterns are suggested as ways to meet healthy eating goals: (1) Healthy U.S.-Style Eating Pattern (H-US), (2) Healthy Mediterranean-Style Eating Pattern (Med), and (3) Healthy Vegetarian Eating Pattern (Veg) [1].

Research using the Healthy Eating Index (HEI), which measures diet quality and adherence to the dietary guidelines, shows that many Americans do not meet healthy eating goals [2,3]. This, in turn, is associated with increased risk of all-cause mortality as well as cardiovascular disease, cancer, and type 2 diabetes mellitus (T2DM) [4]. Additionally, compared to White and Hispanic adults, African American (AA) adults have exhibited greater disparities in diet quality and adherence to dietary guidelines [5]. Similar findings have been reported among US adults with hypertension, T2DM, and obesity [6,7,8].

Social and economic factors such as income, education, access to healthy foods, and inequitable access to healthcare all contribute to disparities in diet quality [9,10,11,12]. There is also an important relationship between food and culture, as food is at the center of the cultural identity and traditions of individuals, thus playing a role in shaping individual eating patterns [13]. Culturally tailored interventions have been effective in promoting weight loss, dietary changes, and improving T2DM outcomes in AA individuals [14,15,16]. For example, AA women who received a culturally tailored workplace dietary interventions saw significant improvements in weight, waist circumference, and weight-related quality of life compared to those who received standard counseling [17]. Another nutrition intervention that tailored the Dietary Approaches to Stop Hypertension (DASH diet) for AA adults led to increased fruit and vegetable consumption and enhanced self-efficacy for healthier eating compared to a control group receiving individual counseling and NHLBI booklets [18]. Thus, identifying cultural values, practices, and beliefs of AA adults may help tailor the USDG dietary patterns to ensure they are culturally relevant and more likely to be adopted.

While numerous culturally tailored dietary interventions for AA adults exist, very few are organized in alignment with the USDG’s 3 dietary patterns [19]. No studies have specifically explored how AA adults interpret and implement the USDG recommendations within their dietary practices. The Dietary Guidelines: 3 Diets study (DG3D) was a randomized dietary intervention study designed to compare the adoption and health outcomes of the three USDG diet patterns among an AA population [20]. The present study aims to explore the acceptability and perceptions of the USDG-based intervention among AA adult participants using USDG materials. This work may inform researchers, educators, and policy makers in the development of culturally relevant guidelines, programs, and materials for AA adults, as well as enhance program adherence, acceptability, and adoption of dietary habits that align with the USDG.

## 2. Materials and Methods

### 2.1. Study Design

This was a qualitative descriptive study embedded within the Dietary Guidelines: 3 Diets (DG3D) randomized controlled feeding trial. The qualitative component aimed to explore the acceptability and perceptions of three USDG dietary patterns (Healthy US, Mediterranean, Vegetarian) among African American adults. A focus group methodology was chosen to elicit shared perspectives and group-level insights following completion of the 12-week DG3D intervention. Data were analyzed thematically using a constant comparative method.

### 2.2. DG3D Intervention

The Dietary Guidelines: 3 Diets study (DG3D) was a 12-week nutrition intervention aiming to assess differences in diet quality (HEI) and T2DM risk factors (weight, Hba1c) among participants (*n* = 63) who were randomized to one of three USDG dietary patterns (H-US, Med, Veg) (ClinicalTrials.gov ID # NCT04981847). The baseline mean HEI score was 58, 55, and 55 for the Veg, Med, and H-US groups, respectively. HEI scores range from 0 to 100 with a higher score reflecting greater alignment with the dietary guidelines. The dietary intervention for all patterns followed USDG recommendations and recipes from MyPlate.gov with no modifications [1] (see Table 1 for an overview of dietary patterns). Participants were encouraged to utilize the MyPlate app, developed by the USDA Food & Nutrition Service, to set daily food goals and earn achievement badges for healthy eating. They also received behavioral strategies from the Diabetes Prevention Program [21]. Participants attended weekly nutrition classes to increase their nutrition knowledge and self-efficacy in following their assigned dietary pattern. Other than the differences across the three dietary patterns, the structure and curriculum for classes were the same for each intervention group. Due to high rates of COVID-19 cases, the study was implemented via Zoom. Classes started September 2021 and comprised discussions, didactic classes (see class topics and their order in an earlier publication [20]), cooking demonstrations, and SMART goals [22], with participants picking up weekly food samples. Classes were led by staff including an AA chef, registered dietitian, undergraduate student volunteers, and a project manager. Participants (83% female; mean age 48.0 ± 10.6 y; BMI 35.9 ± 0.8 kg/m^2^) achieved significant within-group, but not between-group, weight loss (−2.4 to −2.6 kg, *p* = 0.97). No significant between-group differences were found for HbA1c, blood pressure, or HEI, though post hoc analyses showed greater HEI improvement in the Mediterranean group compared to the Vegetarian group (*p* = 0.02). Overall, all three dietary patterns led to significant improvements in weight and diet quality among African American adults. Further information on methods and results has been published elsewhere [20].

### 2.3. Participants

DG3D participants, recruited in summer 2021, had to self-identify as an AA adult, have a BMI between 25 and 49.9 kg/m^2^, live in Columbia, SC, or nearby, and exhibit ≥3 risk factors for T2DM [23]. All DG3D study participants (*n* = 63) were eligible and invited to participate in a focus group discussion (FGD). Announcements requesting volunteers to participate in the FGDs were made during class, and instructions for signing up were provided. Reminders for the FGDs were mentioned during class, as well as through email and phone calls. Of the 63 intervention participants, 42 took part in FGDs (*n* = 17 Med, *n* = 9 Veg and *n* = 16 H-US). Focus groups are more effective when they have 7 to 12 individuals per group; thus, the sample size was more than sufficient for analysis [24]. Participants were given a $20 gift card for their FGD participation. The study received institutional review board approval from the University of South Carolina. All participants provided written informed consent.

### 2.4. Data Collection

Qualitative data were collected during a total of six in-person focus group discussions (two per assigned dietary pattern) conducted in December 2021, upon conclusion of the DG3D intervention. Focus groups were held in private rooms located in the same building where participants attended the orientation and picked up weekly food samples.

The focus group guide was developed by a doctoral student (NO) and principal investigator (GTM) using Social Cognitive Theory [25] and the Designing Culturally Relevant Intervention Development Framework [26]. Social Cognitive Theory guided questions related to self-efficacy, acceptability of diets, and facilitators and barriers to diet adoption. The Designing Culturally Relevant Intervention Development Framework guided questions to help inform the needs for cultural tailoring and examined factors around (a) developmental considerations (e.g., age), (b) cultural considerations (e.g., visual appeal/packaging of the program), and (c) intervention delivery channels (e.g., preferred means of communication). The guide was reviewed with the research team to ensure that questions were clear and that the FGDs would stay within the set time limit. See Supplementary File S1 for the full focus group guide.

The FGDs were facilitated by two trained AA female moderators who were staff members for the DG3D study: NO regularly engaged with participants in weekly classes as a dietitian, while MJW interacted with participants during recruitment, orientation, and pre- and post-intervention laboratory assessments. The focus groups ranged from 50 to 90 min, and sessions were audio recorded and transcribed verbatim by a third-party vendor. Field notes were taken to capture additional nonverbal information and cues.

### 2.5. Data Analysis

Transcripts were thoroughly reviewed for accuracy and analyzed using NVivo version 12 [27] software. Transcripts were coded thematically using a constant comparative method [28,29]. A preliminary codebook was developed from the focus group guide and initial review of the transcripts to guide the initial coding of the data. Additional codes were added iteratively during the analysis to capture emerging themes and subthemes within the data.

To ensure reliability of the analysis and proper interpretation of the data, transcripts were coded by three trained researchers. The researchers coded three transcripts together to ensure coding agreement. The remaining transcripts were each coded by two researchers followed by a group meeting to discuss meaning and interpretation of coded passages and reach consensus on codes and subcodes. Coders also had the flexibility to identify and add new subcodes throughout the process. When new subcodes emerged, researchers went back to previously coded focus groups to see if the new subcodes should be applied.

## 3. Results

Of the 63 participants, 42 took part in the focus group discussions. Participants were all college-educated and included 35 females and 7 males. Six FGDs were conducted, ranging from 4 to 9 participants per group (*n* = 17 Med, *n* = 9 Veg and *n* = 16 H-US). See Supplementary File S2 for participant demographics. Participants shared their experiences and perspectives towards the USDG-based intervention, which are presented under four main themes: (1) perceptions of assigned dietary patterns, (2) barriers and facilitators, (3) cultural relevance, and (4) participants’ recommended changes to the USDG-based intervention.

### 3.1. Perceptions of Assigned USDG Dietary Patterns

As participants were randomly assigned to follow one of three USDG dietary patterns (Mediterranean, Healthy-US, Vegetarian), participants shared their initial perceptions of their diet pattern and how those perceptions may have changed over the course of the intervention.

#### 3.1.1. Mediterranean (Med) Diet Pattern

Those in the Med group perceived their dietary pattern as being “healthy” overall, yet some expressed initial confusion about what constituted a Med diet. Participants in this group reported feeling enthusiastic about being assigned this dietary pattern, as it was seen as a departure from their usual diets, bringing a sense of novelty and excitement. Participants in this group also shared positive feedback about the dietary pattern post-intervention. For example, one participant explained:

“I had heard things about the Med, and to be honest, I thought it was just eating a bunch of olives, tomatoes, and olive oil. Um, but, like, seeing that it still incorporated, like, each of the food groups, just in moderation, I was, you know, pleased.”

Med participant, group 6

#### 3.1.2. Healthy-US (H-US) Diet Pattern

Those in the H-US group reported mixed perspectives about their assigned diet pre- and post-intervention. Most participants initially associated it with a standard American diet, which was viewed as unhealthy and/or too similar to their current dietary pattern. Participants agreed as one shared:

“But I think just historically, when we look at the, the American diet is already in our head, that’s not a good diet.”

H-US participant, group 3

While many participants expressed a desire to try something “*different that we hadn’t been used to*”, some participants found this dietary pattern comforting and doable due to its familiarity. One participant described:

“I think, I know for me, I was glad I got the U.S. diet… I just went grocery shopping and now I don’t really have to change anything.”

H-US participant, group 1

#### 3.1.3. Vegetarian (Veg) Diet Pattern

Those in the Veg group expressed initial skepticism towards following a vegetarian diet. Many reported feeling hesitant about their ability to adopt the dietary pattern. Despite initial hesitancies, participants reported a more positive impression of following the Veg diet pattern post-intervention. A participant described how her first impression after being assigned to the Veg group changed over time by sharing:

“… At first I was disappointed. Just didn’t think I can do it. Never really tried to go without meat, but actually doing it, uh, then I was very proud I was able to do it and I kinda ended up enjoying it. To eat a variety of foods that I would not normally eat, I mean I did eat fruits and vegetables but I guess I was able to eat more and just put the meat out of my mind. So I—I ended up enjoying it at the end.”

Veg participant, group 5

At the same time, participants also reported hopeful assumed associations between vegetarian eating with weight loss. One participant shared:

“So, my first thought and whenever I would tell anybody that I was, you know, on the Veg diet, And the next question would be, well, how much weight did you lose?… You kinda attribute the Veg diet to weight loss. Or that’s kinda what you were hoping to see…”

Veg participant, group 2

### 3.2. Barriers and Facilitators

Participants described their experiences facing various barriers and facilitators to adopting their assigned USDG dietary pattern. These barriers and facilitators were related both to the USDG guidelines and to the overall delivery of the intervention.

#### 3.2.1. Barriers

Three dominant barriers emerged among participant experiences, including dairy avoidance, insufficient meal plans, and family meal preparation. Participants overwhelmingly emphasized the USDG dairy guidelines as the most difficult to meet, as many were either lactose sensitive/intolerant or just did not like dairy products. While the USDG mentions soy products as alternatives, several participants either disliked soy or encountered challenges in finding soy milk. They expressed a desire for the guidelines to incorporate a broader array of dairy-free options, such as almond or oat milk, to meet calcium recommendations. Speaking for many of the participants, one woman stated:

“I think a lot of us had an issue with dairy and getting that in. I love dairy, but I cannot tolerate it as I mentioned before, …. Um, is that something that they could change and be tailored another way we could get calcium that doesn’t include dairy?”

H-US participant, group 3

Some participants found it difficult to come up with meals to prepare that aligned with their assigned dietary pattern. Although they were given a recipe book and a sample meal plan, participants from all diet groups wanted varied examples of meal plans for different meals such as breakfast, lunch, and dinner to make the transition to their dietary pattern easier. One woman suggested:

“The only thing I would say on that is if, if you did that, if you offered some type of, um, sheet, like, one-pager for ideas… Because I know when I started the program I was like, ‘What am I supposed to eat for breakfast?’ And I didn’t know until, like… So, that first week was really confusing to me. Like, what can I eat, um, and still be within plan?… But, at least, like, a week worth of samples. That would be helpful.”

Med participant, group 4

Most participants reported carrying responsibility for the procurement and preparation of food in the household, which especially felt like a barrier to adhering to their dietary pattern when other family members were not receptive to or interested in adopting new dietary choices. One woman described the resistance she faced at home, saying:

“He tried the dishes, didn’t like them (laughing). And he would still eat what he wanted to eat in front of me, so. (laughing). And he kinda laughed when, when I you know told him, ’cause he knew… that our household was a meat household. So, if I had the support that I, that I needed, it would be a whole lot easier. If could’ve gotten everybody on the same uh diet or eating the same meals.”

Veg participant, group 2

Additionally, many participants found it burdensome to prepare separate meals for themselves. For example, one participant shared:

“So I tried a couple of the recipes and they were good, but the issue is I still live at home with my family. So when one of us cooks, we cook for everybody. Um, I live with two picky men and they will not eat pretty much anything there, which makes it difficult…”

H-US participant, group 3

#### 3.2.2. Facilitators

Four dominant facilitators of adopting new dietary patterns emerged among participant experiences, including USDG recipes, nutrition label reading, nutrition class content, and community among participants. See Supplementary File S3 for an overview of selected facilitators to following an assigned USDG dietary pattern.

Many participants enjoyed the recipes from MyPlate.gov as they diversified their cooking. Participants felt the recipes also gave them the opportunity to explore new ingredients like Brussels sprouts and hummus, as well as new dishes such as ratatouille, spinach and fish, chickpea salad, and zucchini stir fry. Some participants further customized the recipes (e.g., adding additional spices) to suit their own preferences. Although some participants expressed initial confusion about how to prepare some of the recipes, participants found the cooking demonstrations of various recipes additionally helpful, as seeing the recipes being prepared piqued their interest and confidence in trying something new. A participant expressed appreciation for recipes that expanded their typical diet by sharing,

“I thought they were, they did well on the recipes in bringing in new ideas and getting us away from the macaroni and cheese and mashed potatoes and rice and gravy.”

H-US participant, group 1

Participants were taught how to read a nutrition label using handouts from the UDDG/MyPlate with emphasis on serving size, calories, nutrition, daily values, and ingredients [27]. Many reported that this activity was helpful in understanding what they were consuming. One participant noted:

“I didn’t realize how much salt, I think we were intaking before I did this study. I knew most of the basics, but just some of the things like saturated fat, salt, even sugars, how much we should have versus how much we probably do have, and just reading the nutrition labels. That was pretty helpful for me.”

H-US participant, group 3

Participants confirmed that the information and activities presented by the staff in the weekly classes, helped them gain knowledge and confidence in their abilities to follow their assigned dietary pattern. Participants reported implementing significant dietary changes, including reduced consumption of butter and sugar, opting for healthier alternatives such as olive oil and honey, a general decrease in sugar and unhealthy snack intake, and increased vegetable intake. Participants also reported a new habit of reading food labels for the sake of “*knowing what the foods consist of*”, and monitoring salt intake, which benefited those managing hypertension. Many described increased mindfulness that led to more home cooking, allowing for more control over ingredients and cost friendliness, as well as increased awareness of food groups and portion sizes. One participant shared the impact of the nutrition classes on her food choices by saying:

“… I thought the study for me was a good thing. It really made me focus and conscious of what I was putting in my mouth and knowing what to avoid …”

Veg participant, group 2

A sense of community among participants was also found to be important to participants’ perceived success. Participants unanimously agreed that the small group discussions recounting the previous week’s successes and challenges were helpful and their favorite part of the class. Participants appreciated hearing about others’ successes and challenges, as it provided encouragement and practical advice and created a supportive environment. Breakout sessions were beneficial as they offered an intimate setting where participants could exchange ideas and realize they were not alone in their journey. This sense of community fostered encouragement and motivation, especially in the absence of in-person interactions. Overall, the participants found that sharing successes and challenges helped them feel supported and empowered as they worked towards their dietary goals together. One participant explained:

“… It kind of feels like a support group seeing as others were able to, um, be successful and not you know, give in and then the times where you were experiencing some type of challenge or someone else is experiencing the same type of challenge and you guys can kind of piggyback of each other’s, um… I enjoyed that, I thought that was beneficial.”

Veg participant, group 5

### 3.3. Cultural Relevance

Participants were asked several questions regarding the perceived cultural relevance of the USDG-based intervention and associated materials for AA populations. While participants generally felt that the dietary patterns and materials were culturally relevant, many shared a desire for increased incorporation of culturally relevant foods and acknowledgment of their community’s influence on dietary habits.

Participants expressed a desire for more staple foods traditionally consumed by AA individuals, like collards, to be incorporated into the USDG recipes provided. One participant mentioned:

“… Like, thinking about some of the things that… African Americans are more accustomed to eat and, like, trying to incorporate those recipes… while still letting us try the new recipes, ‘cause you want us to try different things, but, like, put in those staple items in there for us…”

Med participant, group 6

Many participants mentioned wanting examples of healthier substitutions for cultural dishes and echoed:

“I think that it may be um, helpful culturally to… maybe choose cultural dishes that you know that are popular. And then take that dish and make it Veg or you know easier to you know, or healthier.”

Veg participant, group 2

To make the foods more culturally relevant, participants also expressed a desire for more seasonings in the foods they were instructed to prepare. For example, one participant shared:

“Just seasoning it like we normally would season like to cook it. So if collard greens or something we would either grab some ham hocks or some turkey. But… if you could get the red peppers and the green peppers and don’t put the meat in, just add those and at… just go through your cabinet and put a different kind of seasoning on, you can acquire the taste for it to make it a more cultural dish.”

Veg participant, group 2

As mentioned previously, many participants were sensitive to dairy products. Some participants felt the USDG’s dairy recommendations were not culturally relevant and questioned:

“But I was wondering, does that mean that it’s [USDG] still more tailored to the European diet just because they have, as I’ve read genetically, you know, the genes to be able to process dairy and eat that is pretty much every other minority”

H-US participant, group 3

Overall, participants wanted sociocultural issues to be addressed more comprehensively in the intervention. Some participants emphasized socioeconomic barriers such as limited access and availability of fresh produce and healthy food options, particularly in low-income areas. For example, one participant expressed:

“… I mean you go back in my community you don’t even see a grocery store there. And if you do see a store I mean, it’s a convenience store. Like there is no fresh foods available like that. You just, everything is on shelf, packaged, pre-packaged, you know um, that’s full of carbs. Packed with sodium um, sugar. So in terms of like the reality of it is I don’t know how reali- I mean I don’t, I’m not sure how realistic it is unless you live in like certain kind of communities and you come from like a social, another kinda socio-economical background…”

Veg participant, group 2

Many also mentioned ingrained poor dietary habits, given the prevalence of obesity and fast-food options in their communities.

“Well, I just noticed like, um, as far as our culture, there’s certain things that we tend to eat, what we grow up to eat. Um, we tend to go to fast food places. Um, if you go to a restaurant, and you look around you, you see people that are obese… our community has a high percent of obesity. Um, and we can see it, but we don’t do anything about it. Um, and I just think that it needs to be pointed out more so than it was.”

Med participant group 6

### 3.4. Participants’ Recommended Changes to the DG3D Intervention

As participants discussed the acceptability of the DG3D intervention, multiple prominent suggestions for improvement emerged. Participants recommended making changes to the provided meal plans, virtual class delivery, accountability, social support opportunities, and use of the USDG MyPlate app.

#### 3.4.1. Meal Plans

All participants received a personalized 1-week meal plan detailing recommended servings from each food group, based on the recommended kcal intake for weight loss. The study encouraged a shift away from calorie counting, with staff emphasizing the importance of meeting all food groups and serving sizes. Many participants expressed dissatisfaction with this approach and wanted the intervention materials to emphasize calories for a greater understanding of energy balance and to track participants’ personal weight loss goals. Portion sizes were not perceived as sufficient context to guide healthy food choices. One participant shared her desire for more caloric guidance, saying:

“I mean, it was great talking about the different options, but… how much am I supposed to eat? You know, when I’m looking at calories, how do I look at it when I go in the grocery store?”

Med participant, group 6

#### 3.4.2. Virtual Class Delivery

The use of web-based Zoom delivery for the intervention led some participants to feel less engaged with classes and more susceptible to distraction due to the activities of other family/household members. Many participants thought in-person classes would have led to more interaction with other participants which in turn could have led to increased social support and increased accountability to eat in line with their dietary patterns. Participants resonated with ‘Zoom burnout’ and agreed:

“… People get Zoom burnout, virtual burnout… ’cause we’re on Zoom calls all day. I mean, a lot of us working from home… We’re doing that all day on the computer now. It’s another hour and a half that I’m looking at the computer, someone’s reading. I mean, it’s a lot…”

Med participant, group 6

#### 3.4.3. Accountability

Several participants wanted additional check-ins to promote accountability throughout the study: specifically, weekly tracking of their weight, rather than just pre- and post-intervention. Participants felt consistent weight check-ins would enable them to work toward and keep track of personal goals. For example, one participant shared:

“I think it would’ve been good if, for example, if, if we wanted to lose weight, if we were brought in to say… let’s see what your weight is, let see our progress. I think somebody else says something about our progress too. That would be good too… Just pushing us more.”

H-US participant, group 1

#### 3.4.4. Social Support

Participants expressed a desire for connection with other participants outside of the virtual classes for additional social support outside of classes. Although a Facebook group was provided, Facebook as a platform was not popular among participants with some voicing that a group messaging app would have been better. One participant explained:

“I don’t even have a Facebook page, so that’s why I didn’t participate, ’cause, like, I don’t wanna create a Facebook page just for this, ’cause I probably won’t utilize it. But there was a text, like a group chat text, I would definitely participate in, in that.”

Med participant, group 6

#### 3.4.5. USDG MyPlate App

Most participants did not find the MyPlate app relevant in helping them make food choices. Many participants found the app to be overly simplistic and outdated. Moreover, they expressed dissatisfaction with the absence of a calorie tracking feature, preferring alternative mobile apps that offer this functionality such as Livestrong or MyFitnessPal. Participants reported that the MyPlate app constituted an additional burden as they were asked to utilize it for the duration of the study, and agreed:

“It was just hard for me to focus and keep up with doing that. It was not as helpful. The concept of, you know, what we were supposed to be eating and knowing that was more helpful than me going [to MyPlate app] every day and doing one more thing on my phone.”

Med participant, group 4

## 4. Discussion

This study explored the perceptions of AA adults adhering to unmodified dietary patterns outlined in the USDG (Healthy US, Mediterranean, Vegetarian). Findings provide insights on participant perspectives regarding the 12-week USDG-based DG3D intervention, barriers and facilitators for following the USDG, cultural relevance, and suggested improvements to program implementation. All three dietary patterns resulted in significant weight loss among AA adults who participated in the DG3D study [20], yet due to the prevalence of obesity in this population, it is crucial to understand AA individuals’ experiences with the USDG to implement culturally relevant interventions.

The dietary patterns outlined in the USDG were overall well accepted among the DG3D participants. Veg group participants were not initially excited about their assigned dietary pattern but were more receptive and satisfied post-intervention. Participants listed the weekly classes, classroom activities (e.g., label reading), and sense of community as facilitators to following their diet. Therefore, results suggest the intervention may have helped increase acceptability of the Veg dietary pattern. Studies examining the acceptability of Veg diets among AA populations are lacking. However, one study showed an increase in vegan diet acceptability among all participants post-intervention after both groups were given vegan soul food recipes, and one group given additional vouchers for a vegan soul food restaurant [30].

Participants reported that meeting the dairy recommendations was a challenge. Previous research has also identified similar barriers to dairy intake among AA women, including taste and perceived difficulties with digestion [31]. Lactose intolerance is common among African American individuals [32]; however, reduced dairy intake, leading to lower calcium and vitamin D levels, is associated with chronic diseases that disproportionately affect this population, such as hypertension, metabolic syndrome, and diabetes [33,34]. A nutrition and lifestyle intervention emphasizing calcium intake for AA adults found that educating participants on calcium’s health benefits and introducing non-dairy sources (for example, fortified plant milks) enhanced calcium intake post-intervention [35]. Some AA women obtain more of their calcium from other sources, like grains and fortified cereals [36]. Studies suggest implementing taste-testing of alternative calcium sources, peer education, and group discussion on the importance of calcium as potential strategies to improve calcium intake [37]. Overall, interventions implementing USDG dietary patterns in AA populations should include non-dairy options to meet calcium and vitamin D recommendations.

The USDG and community-based interventions may benefit from awareness and integration of cultural influences on diet patterns and preferences within AA communities. Traditionally, AA religious ceremonies, celebrations, and family gatherings center around food [38]. Common cuisines are typically low in fiber, calcium, potassium, and high in fat and salt/spices, while food preparation tends to involve frying, barbecuing, and adding calorically dense gravies and sauces [13]. Participants indicated a preference for ‘healthier’ versions of traditional and cultural recipes adapted to fit the USDG, as well as familiar seasonings in the recipes. AA participants in a similar study reported that adjustments were needed to a combined DASH (similar to H-US) and Med diet to make it more appealing to AA adults, such as adding familiar staple items and seasonings [39].

Additionally, participants underscored the impact of environmental barriers on eating behaviors, including the abundance of fast food and lack of fresh, affordable produce. Multilevel interventions, rooted in socioecological models, that address structural barriers on eating behaviors within can be employed to create changes at individual, community, and policy levels [40,41]. Establishing farmer’s markets and collaborating with supermarkets and food voucher programs can be a way to increase access to affordable and nutritious foods [42]. For example, a study targeting utilizing a multicomponent environmental intervention including farmer’s markets and a Super Shopper voucher program resulted in increased purchase transactions [43]. Another study established a monthly fruit and vegetable minimarket at a community site to increase access to fresh fruits and vegetables in an area with few produce options [44].

With the abundance of health apps on the market, it may be important to conduct a quality assessment to ensure the MyPlate app meets the needs of its users. The USDG MyPlate app was not a helpful tool for participants due to the lack of a calorie tracking feature and outdated feel of the app. Similarly, among a national survey of health app users, loss of interest was a primary reason for low usage of health apps, as well as dissatisfaction with calorie tracking features [45]. Granted, many health app users tend to be younger, whereas most participants in the present study were over age 50, which may have also been a factor in not wanting to use the MyPlate app [45,46].

Participants strongly desired a specific calorie target and a means to monitor their progress towards that goal. In addition to wanting the MyPlate app to have a calorie tracking feature, and despite receiving a week’s worth of meal plans tailored to meet personalized MyPlate goals, participants also wished to see calories displayed in the USDG materials. Given that eligible participants had overweight or obesity, this feedback was likely motivated by a desire to lose weight. As obesity disproportionately affects AA adults [47] inclusion of calorie tracking may be an important component of nutrition interventions targeting the needs of AA individuals.

The DG3D intervention, originally planned for in-person delivery, was ultimately conducted online. Many participants in this study requested in-person instead of virtual classes due to their desire for increased support outside of classes, accountability with their goals, and communication with peers. A systematic review of web-based interventions for the management of T2DM found that some of the successful approaches applied in virtual interventions included goal setting, personalized coaching, interactive feedback and online peer support groups [48]. Group-based social support may be more difficult to provide in online settings [49]. Group discussion of successes and challenges related to dietary change was identified as a favorite segment among DG3D participants and helped foster a sense of community and support. Future interventions may benefit from incorporating group discussion and avenues for communication as it allows participants to voice challenges, collectively formulate action plans, and receive social support for behavior change [49]. As technology becomes more prevalent in delivering health interventions, virtual implementation can help overcome various barriers to attendance, including transportation and childcare. Future studies may consider exploring the optimal approach to engage participants with diverse preferences, such as a hybrid model combining in-person and virtual sessions, along with strategies to enhance reach, adherence, and social support in web-based delivery formats.

This study provides in-depth perspectives of AA participants, addressing the gap in the limited research on the adoption and acceptability of the USDG diet patterns among AAs. Some limitations may impact the generalizability of findings. Participants were all college-educated, resided only in the southeastern US, and most were women. AA men and younger populations were under-represented. Additionally, barriers experienced by AA individuals in other areas may not be fully represented, such as availability and cost of healthier foods, transportation costs and logistics to procure healthy foods, and economic concerns regarding the perishable nature of fresh foods [50]. Participants volunteered to participate in focus groups, which may reflect a degree of sampling bias due to important systematic differences between those who participated in the focus groups and those who did not [51]. Furthermore, participants were not given the opportunity to comment on transcripts or conduct participant checking of the findings [52]. Also, the focus groups were conducted by staff involved in the intervention delivery and may have influenced biased responses [51]. Lastly, although the present study provides rich qualitative insights into participants’ perceptions and experiences, it does not employ quantitative analyses or statistical comparisons between groups. This limitation reflects the study’s exploratory design, which prioritized depth of understanding and the identification of thematic patterns rather than measurement or hypothesis testing. Future research using mixed-methods or quantitative approaches could build on these findings to examine the prevalence or strength of the identified themes across larger and more diverse samples.

## 5. Future Research Directions

Future studies should explore how the United States Dietary Guidelines can be adapted to better reflect the cultural food practices and preferences of African American adults. Incorporating traditional foods, seasonings, and preparation methods into dietary guidance may improve both the acceptability and sustainability of recommended patterns. Research is also needed to identify and test strategies for increasing calcium intake without relying on dairy products, such as incorporating fortified plant-based alternatives and culturally familiar non-dairy sources.

In addition, future work should examine how multilevel interventions can address structural barriers such as food deserts, limited affordability, and reduced access to fresh produce. Partnerships with community food systems, including farmers’ markets and local retailers, may be particularly effective in expanding access to healthier food options. Technology-based tools also require further evaluation; redesigning mobile health applications to include calorie tracking, personalization, and culturally tailored features could improve usability and engagement among diverse age groups.

Another important avenue for future research is the delivery format of dietary interventions. Comparative studies are needed to evaluate the effectiveness of in-person, virtual, and hybrid models, with particular attention to the role of peer support, accountability, and group discussion in promoting adherence. Finally, expanding the scope of research to include African American men, younger adults, and individuals in different geographic regions will provide more comprehensive insights. Longitudinal studies are also warranted to assess the long-term sustainability of dietary changes and their impact on health outcomes such as diabetes prevention and weight maintenance.

## 6. Conclusions

This study examined the acceptability and perceptions of AA adults adopting unmodified dietary patterns outlined in the USDG (Healthy US, Mediterranean, Vegetarian). Study findings suggest that while participants were able to adopt changes to their dietary patterns over the course of the DG3D intervention, adaptations to the USDG dietary patterns and future dietary interventions are needed to ensure cultural relevance for AA adults in the US. Results from this study can inform future modifications of the USDG as well as behavioral health interventions aiming to improve the health of AA adults.

## Figures and Tables

**Table 1:** Servings size recommendations and food groups for each dietary pattern came from the 2020–2025 Dietary Guidelines for Americans (Table A3-2 H-US, Table A3-4 Veg, Table A3-5 Med in [DietaryGuidelines.gov]) [1].

Dietary Group	Food Group Recommendations
Mediterranean	Emphasize vegetables, fruit, grains, beans, and dairy. Include foods from all food groups.
Vegetarian	Exclude meat products (red meat, fish, poultry) and emphasize plant-based foods like fruits, vegetables, whole grains, legumes/beans, eggs, and low-fat dairy products.
Healthy US	Include low-fat meat, fish, or poultry, low-fat dairy products, fruits, vegetables, whole grains, and legumes/beans.

## Data Availability

The data presented in this study are available on request from the corresponding author due to privacy considerations.

## Supplementary Materials

The following supporting information can be downloaded at: https://www.mdpi.com/article/10.3390/nu17213453/s1, File S1: Focus Groups Interview Guide; File S2: Characteristics for African American participants randomized to follow one of three USDG dietary patterns who participated in focus groups discussion (*n* = 42); File S3: Selected reported facilitators to adopting an assigned USDG diet pattern (*n* = 42).

## Abbreviations

The following abbreviations are used in this manuscript:

AA	African American
DG3D	Diet Guidelines 3 Diet
USDG	United States Dietary Guidelines
USDA	United States Department of Agriculture
H-US diet	Healthy US diet
Med diet	Mediterranean diet
Veg diet	Vegetarian diet
HEI	Healthy Eating Index
T2DM	Type 2 Diabetes Mellitus
DASH	Dietary Approaches to Stop Hypertension
FGD	Focus group discussion
