# Supplementary material for: Perceptions of the Three Dietary Patterns of the 2020–2025 United States Dietary Guidelines Among African American Adults After a 12-Week Randomized Intervention Trial to Reduce Type 2 Diabetes Risk: A Qualitative Study
Source: Nutrients. 2025 Oct 31;17(21):3453. doi: 10.3390/nu17213453 (PMC12608769; doi:10.3390/nu17213453)
Supplement: Supplementary file 1 [file nutrients-17-03453-s001.zip › Supplementary File S1.pdf]

Focus group questions aimed at identifying barriers, facilitators, acceptability, cultural relevance, and recommended changes from African American adults participating in a randomized intervention following USDG dietary patterns (Healthy US, Mediterranean, Vegetarian).

Instructions for moderators: Notes to focus group moderators are listed in bold and should not be read out loud. You will be conducting different focus groups for each diet. Before each focus group, confirm (or have a research assistant confirm) with the participants at check-in that they are there for the correct diet group. Also use a watch as a timer to keep track of time during each section. Once time is up, move on to the next section, but not abruptly.

Introduction:

Good evening, as you already know, my name is [interviewer name], and I'm a research assistant/ the project manager for the DG3D study, and I will be leading our discussion today. Also, we have [notetaker name] here with us who will serve as the notetaker. It's nice to see you all in person after 12 weeks of Zoom classes! You are here because we are interested in learning about your experiences in the program over the last 12 weeks. This will help us continue to improve the program when we offer it to other African American adults who are at risk for type 2 diabetes in our community. As mentioned in class, this focus group will be audio recorded so we don't miss anything that is shared in our discussion. **(Moderator and notetaker press record here).**

Please know there are no right or wrong answers; so, feel free to share openly and honestly. We will be taking notes throughout the discussion. All your identities will remain confidential; your name won't be connected with your responses, and the responses will only be used for research purposes, such as publications or presentations. Also, you may stop taking part in the group discussion at any time. And your participation is voluntary, so you don't have to answer a question if you don't want to. You will receive an electronic $20 Amazon gift card for participating in this focus group, which will be emailed in 1-3 business days.

Your verbal agreement to continue with the group discussion will be your consent to be in the study.

To allow for our conversation to flow more freely. I'd like to go over some ground rules:

1. We'd like everyone to participate actively in the discussion. Everyone doesn't have to share for every question, but I'd like to hear from each of you during this session.

2. Feel free to share as much as you feel comfortable, and please don't share anything said by others during the discussion group with anyone else. This is a judgment-free environment.

3. Only one person speaks at a time. It is difficult to capture everyone's experiences and perspective on our audio recording if multiple voices are speaking at once.

4. Please avoid side conversations so that we can capture all feedback.

5. There are no wrong answers, so please respect the opinion of everyone.

6. Last, since we are all wearing masks, try to speak as clearly as possible so the recordings can pick up what you say.

Do you have any questions before we get started? **Pause here for any questions. (Intro 5minutes)**

<u>SECTION 1</u>: **Introduction and Overall Impressions. (Suggested Pacing: 7minutes)**

Overview: To begin, I'd first like to ask about your overall impressions of the study. **Note to focus group moderators: Let participants respond freely before offering probes. We do not want to prime their responses. Probe as appropriate based on their responses. Suggested probes are included for some of the prompts, but please offer your own probes, particularly when a participant answers with only a few words or if there is something relevant to the topic you'd like to follow up on. Further, feel free to skip around the interview guide based on participant responses.**

1. What made you interested in taking part in the DG3D study?
   Probe: What personal goals did you have when you started this study?

2. Overall, how would you describe your experience with the DG3D study?

3. Before the study, what did you know about the dietary guidelines?

Note to interviewer: Ask for a show of hands. If no one raises their hand, move onto Section 2. If some people have, suggested probes: Had you ever been to the website? Was it similar to what you learned in the study, or different?

<u>SECTION 2</u>: **ACCEPTABILITY AND INTERPRETABILITY OF ASSIGNED DIET. Suggested Pacing: 15min**

Thank you all for sharing. Now I'm going to ask you some more questions about your assigned diet. **Note to focus group moderators: The focus group guide below includes the instructions [assigned diet], please insert the same of the diet assigned to the focus group you are currently conducting.**

1. What were your first thoughts when you found out you were assigned to the **[assigned diet]** group? **Probe:** What did you know about the assigned diet? What types of emotions or feelings did you have when you found out your assignment?
2. Did your opinion about the assigned diet change after completing the program? If yes, how so? **Probe:** What did you learn about the diet that surprised you? What things about the diet were just as you expected?
3. Which of the food groups in MyPlate was most difficult to meet the recommended daily servings and why?
4. What was your experience meeting the dairy group requirements for your diet? **Probe: Do you have any suggestions on how that food group might be revised for an African American audience.**

5. Which food group was easiest to meet the recommended daily servings and why? **Probe based on responses. Why was that food group easier than the food groups that were cited as difficult?**

<u>SECTION 3</u>: ACCCEPTABILITY OF MATERIALS USED AND RECOMMENDED CHANGES. Suggested Pacing: 20 minutes

Thank you. Next, I would like to ask you some questions about the content of the classes. **Hand out a list of classes as needed.**

Reminder of classes for moderator to refresh participant memories if necessary. You don't need to read them out unless they ask or need clarifications.
Class 1: Welcome to the Diet Guidelines Program
Class 2: Introduction to your diet - Breakfast
Class 3: Introduction to your diet - Lunch
Class 4: Grocery Store Tour
Class 5: Have healthy food you enjoy
Class 6: Food Preparation/Stress Management
Class 7: Eat Less Fat and Fewer Calories/Negative Talkback
Class 8: Handling Holidays, Vacations, and Special Events
Class 9: Move those muscles (Physical Activity)
Class 10: Healthy Eating – one meal at a time / Slippery slope to long term lifestyle change
Class 11: Quizzes! / Tip the Calorie Balance
Class 12: Take Charge of What's Around You / Ways to stay motivated

1. Thinking back over the 12-weeks of class, which class or classes stood out to you as being really helpful and why?

Probe: Let participants offer responses freely. If they struggle to recall, remind them of the class topics. If they say 'all were helpful, ask them for their top 1 or 2 classes? Then probe around their experience 'What was so helpful about ____ class?'; What things from ____ class are you using the most day to day?

2. Which class or classes were NOT very helpful and why?
3. What topics would have been helpful to you that were NOT covered in class?

4. What sections of the class were most helpful? Remember that each class reviewed SMART goals from the previous class, had a discussion of successes and challenges, covered a new topic, included a cooking demonstration, and finished with setting SMART goals for the following weeks.

5. What are your thoughts on the order of our class topics? Were there topics you wish we covered earlier in the 12 weeks? **(If they mention adding some classes earlier or later in the program, inquire why)**

6. What did you think about the class length? **Probe: Were classes too long, too short, or about, right? Note to moderators: Give them a little time. If they can't think of anything, offer 'Thinking through your time during the program, was there anything that you were struggling with that hasn't been covered in class?'**

7. Now I'd like to ask you about some of the other course materials. Besides your time in class, we would like to know what materials you found useful for following **[assigned diet]**.

a. **Moderators show the Med and Veg group pyramids from Oldways.** Ask: We only used MyPlate as a guide for your diet. However, there are other food guides that are available that may have provided better guidance for your diet group. Would this have been helpful as a way to guide your food intake for your diet?

b. Did you find the kitchen notes on the DG3D participant website helpful?

c. How much did you use Handouts that were on the participant website? **How culturally appropriate (or relatable to African American food and culture) were these materials? Did they address barriers that were relevant to you?**

d. How much did you use Recipes? **Probe: What are some recipes or types of recipes that you wish were included in the recipes you received from DG3D? Do you think these recipes are suitable for African American audiences? If participants do not freely offer, ask them about their friends and family that are AA. Do they think these materials would be relevant to them?**

e. Did you join the DG3D Facebook group? How much did you use the Facebook group? **Probe if they did not join:** What were some of the reasons why you did not join the Facebook group? **Probe based on response; is the participant active on Facebook more generally? Are there privacy concerns, or concerns about social media sites obtaining personal information? If participant has Facebook and joined the group:** What was most helpful about the Facebook group?

8. How can we improve DG3D to make the **_____**diet more appealing to African American adults?

<u>SECTION 4</u>: BARRIERS AND FACILITATORS OF DIETARY CHANGE. Suggested Pacing: 10minutes

Now, I'd like to ask you about what has made it easier or harder for you to follow this diet.

1. First, how has being in the study changed what you eat? **Suggested probes: Is how you eat at home different from when you are away from home? What, if anything, has changed in your home as you try to adopt this diet?**
2. Overall, how difficult or easy has it been to follow your diet? **Probe: What is the biggest factor that has made it easy? What has made it more difficult?**
3. What role did your family and friends play in helping you or making it harder for you to follow this diet?

Cultural Relevance

1. How culturally appropriate would you say [assigned diet] is for you? **Probes:** How relevant do you think this diet would be to other African Americans and African American communities? What things felt familiar to you about the diet? What things felt uncomfortable? What parts of this diet did you think could blend with or combine with traditional African American dishes.

2. How likely would you be to recommend [assigned diet] to friends and family? **Probe based on responses; you may also use the responses for #4 to shape your probes.**

Thank you for your time. We will be following up with you about your gift card shortly. Please be sure to stop by the kitchen to pick up your meal that [nutrition educator] made.
