# Supplementary material for: Perceptions of the Three Dietary Patterns of the 2020–2025 United States Dietary Guidelines Among African American Adults After a 12-Week Randomized Intervention Trial to Reduce Type 2 Diabetes Risk: A Qualitative Study
Source: Nutrients. 2025 Oct 31;17(21):3453. doi: 10.3390/nu17213453 (PMC12608769; doi:10.3390/nu17213453)
Supplement: Supplementary file 1 [file nutrients-17-03453-s001.zip › Supplementary File S2.pdf]

Characteristics for African American participants randomized to follow one of three USDG dietary patterns who participated in focus groups discussion (n = 42).

Characteristics	
**Age (years), median (range)**	52 (26-65)
**Sex, n (%)**	
Male	7 (17)
Female	35 (83)
**Diet Group, n (%)**	
Healthy US	16 (38)
Mediterranean	17 (41)
Vegetarian	9 (21)
**Body mass index (BMI), mean ± SD**	36 (6)
**Education, n (%)**	
Some college	3 (7)
College graduate	20 (48)
Advanced degree	19 (45)
**Primary Occupation, n (%)**	

Technical, sales, administrative	7 (17)
Professional specialty	11 (26)
Executive, managerial	7 (17)
Service Occupation	2 (5)
Retired	8 (19)
Other	6 (14)
No current employment	1 (2)
**Marital status**, **n (%)**	
Single	9 (21)
Married	22 (52)
Living with partner	1 (2)
Divorced/Separated	10 (24)
