# Supplementary material for: Perceptions of the Three Dietary Patterns of the 2020–2025 United States Dietary Guidelines Among African American Adults After a 12-Week Randomized Intervention Trial to Reduce Type 2 Diabetes Risk: A Qualitative Study
Source: Nutrients. 2025 Oct 31;17(21):3453. doi: 10.3390/nu17213453 (PMC12608769; doi:10.3390/nu17213453)
Supplement: Supplementary file 1 [file nutrients-17-03453-s001.zip › Supplementary File S3.pdf]

Selected reported facilitators to adopting an assigned USDG diet pattern (n = 42).

**Selected Facilitators**	**Representative quotes**
USDG recipes	*"I altered them to my taste in different things. Like the ratatouille she made, I made mine just a little differently, but I did do it. And a couple of them, I did try, and then I just, you know, did my own little flare with her basic information so I think the recipes were definitely helpful."* Med participant, group 4.<br><br>*"I think the recipes were definitely helpful. And there's others that I also tried; that last one she did with the fish and the spinach- That's on my list to do 'cause that was- that was good. That sample was really good."* Med participant, group 4.<br><br>*"...I would've never thought in a million years that I would've ate brussels sprouts but I did... It was really tasty. So I definitely increased my pallet on the different types of vegetables and the way to cook 'em."* Veg participant, group 2<br><br>*"I did the chickpea salad and the zucchini stir fry, I just loved that. I have, um, sliced zucchini and sliced squash in containers in the refrigerator now."* Med participant, group 4.

	"I didn't use any other recipes except for the, um, cooking demonstrations. I did use those... Just to see it, that was helpful for me." Veg participant, group 2
Nutrition classes	*"I used to be a butter person, or put butter in sugar in everything. Since doing this, I kind of backed away from the butter and the sugar. And I've learned not to throw the butter in the pan when I'm making my egg, I'll take the little crystal thing or whatever, and just do a little bit of that. And I use more olive oil now than I've ever used before and sugar wise, I use Splenda and I try not to make my drink as sweet as I would like to have it. So that helped me in this study..."* H-US participant, group 3<br><br>*"I didn't come up with the label reading, I mean that's something different. No matter how many times you go to the grocery store, you sit there, you got to pick up what you want and now I spare a couple of minutes and then I look and read the label and see so that has, you know, been something very different. Knowing what the foods are, you know, consist of."* Veg participant, group 5<br><br>*"I'm more vigilant of salt intake. Everything has salt in it. And I read the labels more now for the salt intake. Especially for high blood pressure."* H-US participant, group 3

	"I think for me, it made me focus more on vegetables. I always eat vegetables but not to the extent that this, um, diet made me really zone in and think about it. Because before, I, you know, you're eating vegetables, but are you eating enough vegetables? So, it just made me hone in on that better. I never paid that much attention to each of the food groups for my meals- So, with this and never havin' been on a diet before, I look at, you know, whether or not I have something in each of the food groups, and um, that's been very helpful for me" Med participant, group 6<br><br>*"I take my lunch that way I can control more of what's in it as well as the portion sizes."* H-US participant, group 3
Sense of community with other participants	*"... the successes and failures kind of help us see that it's not all easy for everyone. You know, and some people might happen to be struggling with the same thing. You say, oh, I'm struggling with that too. How are you handling it? And I think those really, really help. That probably helped more than anything, besides you know learning the stuff we learned. I think that helped more than anything because it was just like, okay, I don't feel alone in my successes, and in my failures. And so that whole sense of community again, is*

	encouraging, especially when we can't meet in person." H-US participant, group 1<br><br>*"...To hear other people's interpretation of what we're supposed to eat. Okay. You know, gave me different ideas of what I could try. And... that we're not alone in this, you know?..."* Med participant, group 6
